# Strategies for the Generation of Gene Modified Avian Models: Advancement in Avian Germline Transmission, Genome Editing, and Applications

**DOI:** 10.3390/genes14040899

**Published:** 2023-04-12

**Authors:** Young-Min Kim, Seung-Je Woo, Jae-Yong Han

**Affiliations:** 1 Avinnogen Co., Ltd., Seoul 08826, Republic of Korea; ypoc01@hanmail.net; 2 Department of Agricultural Biotechnology, Research Institute of Agriculture and Life Sciences, College of Agriculture and Life Sciences, Seoul National University, Seoul 08826, Republic of Korea; sjwoo0818@snu.ac.kr

**Keywords:** avian model, genome editing, primordial germ cell (PGC), CRISPR/Cas9

## Abstract

Avian models are valuable for studies of development and reproduction and have important implications for food production. Rapid advances in genome-editing technologies have enabled the establishment of avian species as unique agricultural, industrial, disease-resistant, and pharmaceutical models. The direct introduction of genome-editing tools, such as the clustered regularly interspaced short palindromic repeats (CRISPR) system, into early embryos has been achieved in various animal taxa. However, in birds, the introduction of the CRISPR system into primordial germ cells (PGCs), a germline-competent stem cell, is considered a much more reliable approach for the development of genome-edited models. After genome editing, PGCs are transplanted into the embryo to establish germline chimera, which are crossed to produce genome-edited birds. In addition, various methods, including delivery by liposomal and viral vectors, have been employed for gene editing in vivo. Genome-edited birds have wide applications in bio-pharmaceutical production and as models for disease resistance and biological research. In conclusion, the application of the CRISPR system to avian PGCs is an efficient approach for the production of genome-edited birds and transgenic avian models.

## 1. Introduction

Although the chicken was the first organism to have a sequenced genome after the Human Genome Project [1], it is not a well-established model for genome editing. Avian model species, such as chickens, have unique developmental characteristics, in which the fertilized embryo grows in the eggshell until hatching, thus enabling easy access and manipulation of specific embryonic stages [2]. The avian-specific ex vivo research system can be used to directly monitor cell differentiation, transformation, and organogenesis, and contributes to basic and clinical research. Recent advances in genome modification technologies, such as the clustered regularly interspaced short palindromic repeats (CRISPR)/CRISPR-associated (Cas) system and transgenic systems, have substantially expanded the applications of genome-edited avian models in various fields, including agriculture, healthcare, and disease control [3,4,5].

The chicken is a particularly advantageous model for recombinant protein production because chicken eggs contain about 3.5 g of egg white protein with similar glycosylation residues to those of human and target proteins are easily purified compared with other animal bioreactor systems [6,7]. Theoretically, if the protein composition of proteins is altered by genome-editing technologies, chickens could be a promising animal bioreactor system. Moreover, the risk of exposure to exogenous contaminants and the cost of feeding a hen are relatively low, enabling large-scale recombinant protein production. Therefore, in the poultry industry, there is substantial interest in the use of genome-editing technologies to efficiently produce recombinant proteins with applications in various fields, such as medicine. Furthermore, there is a focus on the development of disease-resistant lines to minimize economic losses in the poultry industry [8]. There are about 23 billion chickens, which is about 5 times more than the estimate 50 years ago, reflecting the dramatic increase in the poultry industry and market over the past few decades [9]. This trend is expected to continue, with corresponding increases in demand for eggs and meat. In this regard, disease control in the poultry industry is needed. In particular, avian influenza viruses have severely threatened poultry farms and caused enormous economic losses [10]. Therefore, the demand for genome-edited avian models with resistance to various poultry diseases is increasing.

Germline genome modification is essential to produce genome-edited avian model. In general, modification of totipotent embryo stem cells followed by transplantation into recipient embryos can produce transgenic mammals [11]. In addition, somatic cell nuclear transfer (SCNT) was adopted for transgenesis in mammalian species; however, physiological differences (discussed below) limit the application of these approaches to avian species. Although long-term inducible gene silencing by small hairpin RNA (shRNA) was demonstrated in chicken embryos [12], avian genome modification substantially expanded the scope of research on gene function and enabled the development of genome-edited avian models with multiple applications. In this regard, studies of germline-competent stem cells of avian species have focused on model development by genetic modification and genome editing [13]. This review provides an overview of genome editing in avian taxa and discusses the use of germline-competent cells to develop genome-edited avian models.

## 2. Historical Overview of Genome Modification Strategies for Avian Model Development

### 2.1. Overview of Avian Transgenesis

In initial techniques for mouse transgenesis, foreign genes from viral DNA were successfully introduced in the host genome by microinjection into the pronuclei of fertilized oocytes [14]. Putative genome-modified embryos were implanted in the oviducts of surrogate females, and the resulting progeny contained the foreign gene [14]. This approach was subsequently attempted in rabbit, livestock (sheep and pigs) [15], sea urchins [16], frogs [17], and flies [18,19]. This approach has been the most common transgenic and genome-edited animal production method for decades [20]. Although introducing foreign genes into fertilized eggs from various species is a well-established routine method for transgenesis, its application to birds has been limited. Unlike mammalian eggs, it is hard to obtain single chicken embryos and to manipulate the fertilized chicken embryos located within the eggshell and vitelline membrane, which is not conducive to microscopy. It is also difficult to introduce foreign genes, since the pronuclei of chicken eggs are surrounded by the opaque yolk-filled cytoplasm, and numerous sperm nuclei exist on the membrane [21,22,23]. Moreover, the Eyal–Giladi and Kochav (EGK) stage X blastoderms [24,25,26] of freshly laid eggs already consist of numerous cells (approximately 40,000–60,000 cells), and there are around 50–100 primordial germ cells (PGCs) until oviposition (EGK V-EGK X) [27,28]. This indicates that embryonic development progressed considerably before oviposition, distinct from fertilized eggs of mammals. Despite the production of transgenic birds by the microinjection of linearized DNA constructs in chicken zygotes, the practical application or feasibility of this method is limited by the complexity and low production rate [29].

### 2.2. Virus-Mediated Gene Insertion for Avian Transgenesis

An alternative to conventional foreign DNA transfer systems for bird transgenesis, virus-mediated gene transfer, has been established. The first transgenic chicken was established by transduction of EGK stage X blastoderms with a replication-competent retroviral system based on the avian leucosis virus (ALV) [30,31]. However, the germline transmission efficiency was too low to merit its application to further transgenic line development. In 1989, Bosselman et al. developed a replication-defective reticuloendotheliosis virus (REV) vector-mediated germline transgenic chicken system, including genes encoding thymidine kinase and neomycin phosphotransferase [32]. The replication-defective system is more widely and commercially available than the previous replication-competent system [30]; however, the germline transmission efficiency is still very low (approximately 8%) [32]. A replication-deficient retrovirus based on ALV was further applied to chickens to develop a transgenic bioreactor system. Harvey and colleagues have demonstrated β-lactamase production in chicken egg whites and serum using the ubiquitous cytomegalovirus (CMV) promoter [33]. Around this time, more effective viral vector systems were developed for transgenic chicken production. The lentiviral system uses a genus of retroviruses that can infect dividing cells, thus enabling transgene introduction into somatic and germ cells with a higher efficiency compared with that of the previous retroviral system [34]. The germline transmission efficiency varies from 4% to 45%; however, the frequency is comparatively higher than those of other retroviral systems. Recently, adenovirus-mediated CRISPR/Cas9 genome editing in chicken blastoderm PGCs produced genome-edited offspring with 11% germline transmission efficiency [35].

Similarly, virus-mediated gene transfer has been established in other avian species. For example, transgenic quails have been developed using a retroviral vector based on Moloney murine leukemia virus (MoMLV) pseudotyped with vesicular stomatitis virus, with high germline transmission efficiencies (around 80%) [36]. A lentivirus system has also been adopted to generate transgenic quails with Rous sarcoma virus (RSV) promoter-driven *EGFP*. Direct transgene introduction into gonadal PGCs achieves germline transmission, although the transmission rate is low (1.6–1.9%) [37]. The lentivirus system has also been introduced to generate transgenic zebra finches (*Taeniopygia guttata*), a representative animal model of vocal learning. After the introduction of a lentivirus containing a human ubiquitin-C promoter-driven GFP transgene into very early stage embryos, 13% (3/23) of founders were germline transgenics [38]. Similarly, a transgenic zebra finch model in which cAMP response element binding protein (CREB) is regulated or expressed in the human mutant huntingtin (*mHTT*) gene was also developed using the lentiviral system to monitor vocal learning behavior and to model Huntington’s disease [39,40]. Recently, embryonic zebra finch PGCs were successfully expanded in vitro for 15 days and the *GFP* gene was inserted by lentiviral transfer. The genome-modified cultured PGCs were used to generate transgenic zebra finch [41]. In addition, an adenovirus containing CRISPR/Cas9 targeting for melanophilin (*MLPH*) gene was injected into the quail blastoderm to produce transgenic quail with 45% germline chimera production efficiency and 2.4% to 10% germline transmission efficiency [42]. Similarly, adenovirus containing CRISPR/Cas9 was injected into duck blastoderms, producing genome-edited progenies with 2% (duck) germline efficiency [35]. Despite these successful cases, viral systems are difficult to regard as an effective method owing to the variation in transgenic production efficiency and the limited precision of gene editing.

### 2.3. Development of an Avian PGC Culture System and Transgenic Models

In most animal species, including the fly, fish, mouse, and chicken, germ cells are specified in the early embryo and migrate to the genital ridge [43,44,45,46,47]. However, the detailed processes, including the origin and migratory route of cells, vary between taxa. In the mouse system, PGCs, the precursor cells of gametes, are first detectable as cell clusters in the proximal epiblast region, then migrate to the extra-embryonic ectoderm (ExE) [48,49], followed by movement through the developing endodermal hindgut into the genital ridges [43]. In the avian system, different from mammals, PGCs are initially detected in a scattered pattern in the area pellucida, the central region of the blastoderm at EGK stage X of freshly laid fertilized eggs [24,26,50]. PGCs localized in EGK stage X move to the germinal crescent at Hamburger and Hamilton (HH) stage 4 [51,52,53]. PGCs enter embryonic blood vessels and circulate through the bloodstream between HH stages 9 and 12 [54,55] and finally settle in the genital ridge [55,56]. The distinct patterns of avian PGC development and migration [57] allow the easy isolation of PGCs at several developmental stages, including the germinal crescent of the embryo, blood vessels, and genital ridge [58]. In particular, avian PGCs from the chicken, pheasant, quail, turkey, duck, and guinea fowl have been isolated by density gradient centrifugation and size-dependent isolation methods, without requiring specific antibodies [59,60,61,62,63,64]. Antibody-mediated methods, such as fluorescence-activated cell sorting and magnetic-activated cell sorting, have also been developed to isolate avian PGCs [63,65,66,67,68].

PGCs derived from embryos have been directly introduced into recipient embryos to restore wild or endangered birds [69,70,71,72,73,74]; however, these previous studies are largely dependent on the chicken embryo as a recipient due to the requirement for the long-term cultivation of PGCs. Among vertebrates, the chicken is the only species in which a long-term PGC culture system has been successfully established and used to produce transgenic animals [75]. For the proliferation and survival of chicken PGCs, basic fibroblast growth factor (bFGF) is required [76,77] and stem cell factor (SCF) or leukemia inhibitory factor (LIF) [75]. When bFGF is added to the culture medium, PGCs can be propagated by the activation of the mitogen-activated protein kinase (MEK)/extracellular signal-regulated kinase (ERK) signaling pathway, maintaining their key characteristics, such as migratory activity and germline transmission, even after long-term cultivation [76,77]. Subsequent studies have suggested that the MEK1, AKT (also known as protein kinase B, PKB), SMAD family member 3 (SMAD3), and Wnt/β-catenin signaling promote PGC proliferation [78,79]. The long-term cultivation of PGCs has also been demonstrated for other avian species. Transforming growth factor β (TGF-β) activated by activin or inducer of definitive endoderm 1 (IDE1) for quail PGC proliferation is effective for in vitro culture for 40–50 days [80]. Gonadal PGCs in the zebra finch have been cultured for 30 days in vitro [81]. Circulating PGCs and gonadal PGCs of the Muscovy duck (*Cairina moschata*), the Pekin duck (*Anas platyrhynchos*) and hybrid mule duck (*C. moschata* × *A. platyrhynchos*) have been cultured for days in vitro in chemically defined medium [64]. These findings provide a basis for the production of transgenic models by manipulating PGCs in several avian species, such as quail [37] and zebra finch [41]; however, reliable long-term PGC culture systems are lacking for species other than chickens.

Since the establishment of long-term cultivation methods, a PGC-mediated transgenic chicken production system has been introduced [75]. The transposon system is an efficient gene delivery system for chicken PGCs [82,83,84]. Incorporation of the *EGFP* gene containing the *piggyBac* transposon into the genome of chicken PGCs after long-term culture results in a high transmission rate in offspring (over 90%). This represents a stable transgenic chicken production strategy, different from previous systems [82]. Transposon systems are easy to produce and inexpensive to purify, with non-immunogenic characteristics compared with virus-mediated systems [85,86]; accordingly, they are considered far more practical and reliable for the PGC-mediated production of transgenic chickens. This technique has been adopted to produce recombinant bioactive protein, human epidermal growth factor (EGF) [87], and human anti-CD20 monoclonal antibody in transgenic egg whites as animal bioreactors [88].

### 2.4. Development of Efficient Germline Chimeras by Depleting Endogenous PGCs for Avian Transgenesis

Cultured PGCs could be gene-edited by a programmable gene editing system (discussed in Section 3) and microinjected into the blood vessels of recipient embryos. The recipient embryos contain both endogenous gametes and donor-derived gametes and are referred to as “germline chimeras”. In 1976, turkey PGCs were injected into the blood vessels of chicken embryo and functional gametes derived from turkey PGCs were produced [89]. Subsequently, quail PGCs were successfully transferred to recipient embryos to produce quail germline chimeras [90]. Using this strategy, the first transgenic bird was produced via PGCs isolated from the germinal crescent of HH stage 5 chicken embryos [91]. In addition, isolated PGCs obtained by density gradient centrifugation and magnetic-activated cell sorting were transferred into a recipient embryo for the efficient production of germline chimeras [66,92]. Germline chimeras were also produced using the cryopreserved PGCs [93,94]. In summary, avian germline chimeras can be produced by microinjecting PGCs isolated from the blood of HH stage 14–16 embryos and gonads of HH stage 26–28 embryos in chicken and quail.

However, a major challenge to increasing germline transmission efficiency is competing endogenous PGCs of recipient embryos. One strategy to enhance germline chimerism is transplanting genome-edited PGCs into the adult testis to obtain functional sperm cells [95]. Another solution is depleting endogenous PGCs of recipient embryos by exposure to γ rays [96] and the elimination of blood from HH stage 14–15 recipient embryos [93]. In 2010, Nakamura et al. showed that busulfan treatment in recipient embryos depleted endogenous PGCs and resulted in a germline chimera efficiency of approximately 99%, compared with an efficiency of only 6% in an untreated group [97]. Although the effect of busulfan treatment could depend on the administration route, time point, and dosage [98], the depletion of endogenous PGCs using busulfan can promote the establishment of efficient germline chimeras and genome-edited birds. Recently, Kim et al. developed an in vivo selection model to increase the efficiency of transgenic chicken production by introducing microsomal glutathione-S-transferase II (*MGSTII*) into the PGCs to confer resistance to busulfan. The *MGSTII*-expressing PGCs were dominantly localized in the recipient testes after busulfan treatment compared to non-treated group. The rate of donor PGC-derived progeny production was 94.68% and the rate of transgenic chicken production was 80.33–95.23%, compared with 51.18% in the group not treated with busulfan [99]. A similar system was applied to zebra finch; *MGSTII*-expressing PGCs were enriched and underwent spermatogenesis in the recipient zebra finch testis [100]. In addition, the chicken DEAD-Box Helicase 4 (*DDX4*) gene, essential for formation of germ cell lineage formation, was disrupted by transcription activator-like effector nucleases (TALENs), resulting in the loss of PGCs and infertile hens [101]. Furthermore, the inducible caspase-9 (iCaspase9), conditionally activated in response to the chemical compound AP20187 (B/B), was combined with Deleted In Azoospermia Like (*DAZL*) for germ cell-specific expression; injecting AP20187 (B/B) conditionally inhibited the growth of endogenous iCaspase9-expressing PGCs, providing an alternative strategy to deplete endogenous PGCs for efficient germline chimera production [102].

### 2.5. Transgenic Systems Using Other Germline-Competent Stem Cells in Avian Species

Pluripotent stem cells, such as embryonic stem cells (ESCs), have been used to produce transgenic mammals [103,104,105,106]. In this regard, the establishment of pluripotent stem cells from undifferentiated blastodermal cells at EGK stage X and of germline chimeras by injecting the stem cells into the subgerminal cavity of recipient embryos has been described in avian species [96,107,108,109]. Culture conditions for chicken ESCs include LIF, bFGF, SCF, and insulin growth factor 1 (IGF-1), which are similar to the chemokines required for mammalian pluripotent stem cells [109]. These cells contribute to all three germ layers and somatic tissues, whereas their germline contribution is lacking or very limited [109,110]. Although the ectopic overexpression of chicken vasa homolog (*CVH*) on chicken ESCs increases germline markers, the contribution to the germline is still unknown [111]. A method for the direct reprogramming of somatic cells into a pluripotent state, or induced pluripotent stem cells (iPSCs), using several transcription factors, has been introduced [112]. This system has been rapidly applied to several species, including humans, monkeys, and pigs [113,114,115]. In avian species, the induction of pluripotent stem cells from embryonic fibroblast cells and feather follicle cells have been demonstrated in the chicken, quail, and zebra finch [116,117,118]. Recently, chicken embryonic fibroblasts (CEFs) were reprogrammed into iPSCs and further induced into PGCs, producing somatic cell-derived offspring [119]. However, more reproducible results for iPSC-mediated viable germline-competent stem cell development (e.g., induced PGCs) are needed in the future.

Germline stem cells or spermatogonial stem cells (SSCs) are also frequently used for transgenic research [120,121,122]. SSCs are reliable germline-competent stem cells able to deliver genetic information to successive generations. In rats, SSC transfection via lentiviral vectors has been used to generate genome-modified progeny after xenografting into heterologous testes [120,121,122]. Recently, this strategy has been successfully applied to other species, such as the tree shrew [123]. In addition, using SSCs, transposable element-mediated gene knockout and genome editing by homologous recombination have been successfully demonstrated [124,125]. However, SSCs only account for approximately 0.03% of germs cells in the mammalian testis. Therefore, SSC enrichment is important for the production of transgenic animals by transgenic cell transplantation [126]. Owing to the scarce availability of SSCs in the testes, efficient methods for their isolation and subsequent enrichment are needed. SSCs have been successfully isolated and cultured from many species, including humans, mice, cattle, and pigs [127,128,129,130]. Chicken and quail SSCs have been isolated and maintained over short durations in vitro [131,132]. More recently, quail SSCs were efficiently enriched by density gradient centrifugation using Ficoll-Paque PLUS (Ficoll) and transplanted into the recipient. The rate of germline transmission by SSC transplantation in germline chimera was 0–13.2% [59]. However, studies of SSCs in poultry and their utilization are still limited. Therefore, SSC-mediated genome-edited avian production requires more research on cell proliferation and gene regulatory systems. Collectively, the production of avian germline chimeras and transgenic animals using pluripotent stem cells or germline stem cells is not yet a reliable strategy compared with the PGC-mediated system.

## 3. Progression of Precise Genome-Editing Systems for Avian Taxa

### 3.1. Programmable Genome Editing in the Avian System

Targeted gene modifications at specific loci have broad implications, including therapeutic applications, implications in the areas of immunity, disease control, neuroscience, developmental biology, and agriculture [133,134,135,136,137]. In mammalian species, genome-edited production systems for the loss- and gain-of-function of loci that direct gene transfer and genome-edited ESCs into fertile eggs have been used for a long time [103,104,105,106]. Unlike in mammalian species, the absence of a reliable transgenesis system has prevented specific gene-targeting in birds until the development of PGC-mediated transgenesis. The first targeted gene knockout in chickens was achieved by homologous recombination in the immunoglobulin gene [138]. The homozygous knockout of Ig heavy chain (IgH) in chickens resulted in a lack of an antibody response after immunization. Despite the successful knockout, the homologous recombination efficiency in the PGC genome was extremely low (approximately 0.1%) because the method depends only on the homology of the template gene, limiting its practical implementation [139]. Thus, developing an efficient programmable genome editing system is a prerequisite for the efficient production of genome-edited birds.

In the last few decades, improvements in our understanding of the nuclease-based bacterial immune system have enabled the development of programmable genome editing, including targeted gene deletions and insertions, and the modification of host genomes [140]. Zinc-finger nucleases (ZFNs) and TALENs are chimeric nucleases comprising site-specific DNA-binding modules with DNA nucleases. These nuclease systems induce genome modifications via double-strand DNA breaks, resulting in error-prone non-homologous end joining (NHEJ) or homology-directed repair (HDR) at the target genome sequence [141]. In 2014, the first TALEN-mediated targeted deletion in chickens, targeting ovalbumin (*OV*), was reported [142]. The genome deletion efficiency for PGCs was up to 33.3%, which is a dramatic increase over that of homologous recombination [138]. The germline efficiency ranged from 22.3% to 53.2%, and the rate of *OV* mutant offspring was around 8% [142]. Since the development of TALEN-mediated genome editing in chickens, the TALEN system has been used for targeted *GFP* gene insertion with HDR repair in the chicken *DDX4* gene, a representative germ cell marker [101]. The development of these programmable gene-editing technologies has made it possible to produce sustainable and reliable genome-edited chicken models.

### 3.2. CRISPR-Mediated Genome Editing in the Avian System

The recent development of the CRISPR/Cas9 system for gene editing is a major advancement; the system consists of two sets of RNA, 20 bp CRISPR RNA (crRNA) and universal trans-activating crRNA (tracrRNA), and a nuclease from *Streptococcus pyogenes* type II, Cas9 protein (Cas9), which induces the cleavage of target loci [143]. This CRISPR/Cas9 system can also edit targeted sequences via HDR or NHEJ, similar to other programmable gene-targeting systems, ZFN and TALEN [144]. Since its development, the CRISPR/Cas9 system has been applied to a wide range of taxa and has quickly become accepted as the most advanced and simple gene-editing system to date [145,146,147,148,149].

The PGC-mediated germline transmission system led to the development of genome-edited chickens via the CRISPR/Cas9 system, similar to the previously described method for transgenic bird production and the CRISPR/Cas9-mediated genome-editing system has been effectively applied to avian species for broad applications. [150,151]. Oishi et al. specifically knocked out chicken *OV* and ovomucoid (*OVM*), which are major egg allergens, in the genome of PGCs using the CRISPR/Cas9 system. CRISPR/Cas9-mediated targeted gene insertion in the chicken IgH variable region (V region) was reported at about the same time [150]. In particular, double-strand breaks were induced at the target site using the CRISPR/Cas9 system, followed by target gene insertion by homologous recombination. Surprisingly, the recombination efficiency was dramatically improved (to 9%) [151] compared with the efficiency of conventional homologous recombination methods for chicken PGCs [138]. In addition, genome-edited chicken with an increased muscle mass was generated by targeting the myostatin (*MSTN*) gene, which controls tissue growth and development, and the G0/G1 switch gene (*G0S2*), which regulates fat deposition [152,153]. In a similar study, recombinant adenovirus containing CRISPR/Cas9 was injected into the quail blastoderm, resulting in significant increases in body weight and muscle mass in homozygous mutants with the deletion of cysteine 42 in the *MSTN* gene [154]. Similarly, the targeted deletion of quail melanophilin (*MLPH*) was demonstrated.

CRISPR/Cas9-mediated in vivo genome editing in avian species has also been introduced. Cas9-expressing chickens showed successful in vivo gene disruption in lymphocytes and embryonic brains [155]. In addition, Challagulla et al. produced interferon α and β receptor subunit (*IFAR1*) knockout chicken by directly injecting transposon vector encoding guide RNAs (gRNAs) targeting chicken *IFAR1* and high-fidelity Cas9 [156]. These results indicate that in vivo genome editing could be used to produce genome edited birds which lack the long term PGC culture method. Collectively, the CRISPR/Cas9-mediated genome editing system considerably increased the efficiency of gene-edited birds’ production; additional applications will be discussed in Section 4.

Although genome editing in animals represents a major advance, its low efficiency (<10%) needs to be resolved before it is considered a practical method [42]. Strategies aimed at increasing gene editing efficiency and accuracy are needed. Unlike the CRISPR/Cas9 system, the base editing (BE) system uses catalytically deactivated Cas9 (dCas9) or nickase Cas9 (nCas9) and cytidine deaminase, which can induce a C-to-T (or G-to-A) substitution, and thereby edit specific nucleotide sequences without double-strand breaks in DNA [157]. The BE system was successfully applied in chicken to induce mutations in *MSTN* and ovotransferrin (*TF*) and 35.7% and 55.5% of genome-edited progenies harbored the desired base substitution in *TF* and *MSTN*, respectively [158]. More recently, a prime editing guide RNA (pegRNA) was developed and Cas9 was fused with a reverse transcriptase (RT) domain to form a programmable nuclease, termed prime editing, able to remove, replace, and insert target sequences in the genome [159]. Prime editing was recently applied to the PGC genome. PEmax and ZsGreen1 were integrated into the PGCs genome by the transposon system and cells were transfected with several candidate pegRNAs inducing a stop codon in *DDX4*. Approximately 8.3% of prime edited PGCs showed the desired substitution in *DDX4* [160]. Based on these studies, advanced genome editing systems are expected to be useful for the production of genome-edited birds in the near future.

## 4. Potential Applications of Genome Editing for Avian Model Development

**Figure 1:**
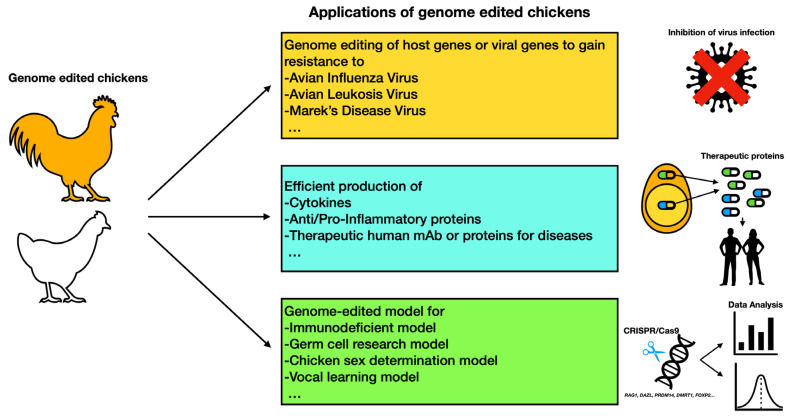
Representative applications of genome edited chickens. The representative examples of genome edited birds introduced in this article are shown. The viruses must use host proteins to successfully complete their life cycle and the host gene edited birds are resistant to various types of viruses, including avian influenza viruses, avian leukosis viruses and Marek’s disease viruses. The target gene encoding therapeutic proteins are inserted into the chicken genome, such as the *ovalbumin* gene, to produce avian bioreactor. The egg white contains a high amount of target proteins that can be used for medical purpose. The specific gene knockout or gene tagging in the chicken provided suitable model for researchers to study the gene function.

The advancement of avian germline transmission and genome editing technology enabled researchers to develop various genome edited avian models, including a disease resistant model, efficient bioreactor, and academic model for scientific use (Figure 1). In the following sections, we will discuss various examples of genome modified avian models.

### 4.1. Genome Editing for the Development of Disease-Resistant Avian Models

Disease control and prevention in birds is an essential prerequisite for the sustainable poultry industry. In this regard, genome-editing technologies have been used to control avian diseases, such as avian influenza, Marek’s disease, and avian leukosis [161,162,163]. In some cases, the effective prevention of avian viral diseases has been achieved by the regulation of virus-specific receptors. For example, a subgroup of ALV can lead to cancer in chickens, and this could be prevented by the regulation of host specific ALV receptors [164]. The precise genome editing of chicken *NHE1*, *TVA*, *TVB*, and *TVC* (specific receptors of ALV subgroups ALV-J, ALV-A, ALV-B, and ALV-C) has been achieved in the chicken DF1 cell line [165,166,167,168]. Such gene editing effectively reduces viral infection, leading to the development of genome-edited chickens. Beyond the cell level, tryptophan 38 (W38) of NHE1, a critical residue for ALV-J entry, was precisely deleted in a commercial chicken line to produce ALV-J-resistant chicken [169].

In relation to avian influenza virus (AIV), resulting in high mortality rates in birds, the host factor *ANP32A*, which supports the vPol activity of influenza A virus (IAV) in a species-specific manner, is critical [170,171]. The 99 nucleotides of chicken *ANP32A* encoding the additional 33 amino acids in birds have been deleted in chicken DF1 cells and viral polymerase (vPol) activity was significantly reduced [162,170,171]. Recently, the critical residues (aspartate 149 and 152) for interactions with viral protein in the additional 33 amino acids of chicken ANP32A were revealed [172]. In addition, chickens lost Retinoic inducible gene 1 (*RIG-I*), a major IAV RNA sensor in mammals; they only harbor Melanoma differentiation associated protein 5 (*MDA5*), a member of RIG-I like receptor (RLR) family [173]. To gain resistance against IAV, duck *RIG-I* was introduced into the chicken DF-1 cells [173,174]. Furthermore, the chicken MDA5 C terminal domain (CTD) was replaced with that of RIG-I, resulting in greater inhibition of viral proliferation than that of wild-type chicken MDA5 [175]. Genome editing at these loci could be an effective strategy for the control of avian influenza in a host-specific manner.

Another model of avian disease control has also been proposed for Marek’s disease viruses (MDVs), a lymphotropic α-herpesvirus associated with T-cell lymphoma that induces asymmetric paralysis of the limbs, depression, and death. The targeted disruption of genes essential for MDV replication suggests that effective disease control is possible [163]. Recently, transgenic chickens expressing both Cas9 and gRNA specific to the immediate early infected-cell polypeptide-4 (ICP4) of MDV were produced. The chicken embryonic fibroblasts from transgenic chickens inhibited MDV infection with no effect on herpesvirus of turkeys (HVT) infection [176]. Based on these results, an in-depth understanding of the infection route, replication pathways, and host–virus interactions will provide a basis for the development of an effective genome editing-based disease control model in avian species.

### 4.2. Practical Bioreactor System for Recombinant Protein Production in Avian Systems

Transgenic avian bioreactors have great potential for recombinant protein production, including proteins with pharmaceutical and industrial applications in eggs [177,178,179]. Several transgenic avian bioreactors have been reported [88,180,181,182]. However, the conventional system is limited with respect to the quantity of recombinant protein produced. Still, the CRISPR/Cas9 system has made it possible to develop an effective chicken bioreactor system for large-scale production in eggs. Oishi et al. inserted human interferon-β (IFN-β) into the chicken *ovalbumin* gene via the CRISPR/Cas9 system to produce high levels of recombinant protein (3.5 mg/mL) in chicken egg whites [183]. Human interferon α 2a and porcine colony stimulating factor 1 (CSF1) fused with the Fc region was produced in transgenic chicken eggs and recombinant proteins produced from the chicken bioreactor were easily purified and showed comparable biological functions to those of recombinant proteins produced by other systems [6]. Kim et al. produced anti-cancer monoclonal antibodies against the CD20 with greater Fc effector function in chicken egg whites compared to a commercial counterpart [88]. In addition, gene encoding adiponectin (ADPN), a hormone derived from adipose tissue that can be used to treat insulin resistance, was precisely integrated into the *Ovalbumin* (*OVA*) by CRISPR/Cas9 system. The gene-edited chicken expressed high amount of high-molecular-weight (HMW) ADPN, considered to be a more active form [184]. Recently, the *GFP* gene was inserted into chicken ovalbumin (*OVA*) gene and system for evaluating protein production in a chicken bioreactor using young chicks was established. This system measured GFP expression in the oviduct of 3-week-old chicks after treatment with an estrogen agonist, diethylstilbestrol (DES) [185]. These results provide a basis for the development of an ideal animal bioreactor that overcomes issues related to yield.

Recombinant proteins from the chicken oviduct derived from egg bioreactor systems with unique post-translational modifications related to *N*-glycan species terminated with high mannose with a core afucosylated form have been reported [180]. Based on these characteristics, an enzyme produced by transgenic chickens was developed for enzyme replacement therapy for Gaucher disease [186]. As enzymes for the treatment of lysosomal storage diseases, including Gaucher disease, Pompe disease, and Fabry disease, are taken up by mannose receptors, the terminal mannosylation of these recombinant enzymes is critical for efficacy [187,188,189,190]. In this respect, recombinant proteins derived from chicken fallopian tubes are suitable bioreactors to produce these enzymes. Alternatively, the afucosylation of recombinant proteins produced in transgenic chickens can be an effective system for the production of anti-cancer antibodies. *N*-glycan afucosylation of the Fc domain affects the antibody-dependent cellular cytotoxicity of therapeutic antibodies [191]. Accordingly, levels of antibody-dependent cellular cytotoxicity of antibodies derived from transgenic chickens are significantly higher than those of control chickens [88,180]. Overall, an effective genome-edited avian bioreactor model obtained by CRISPR/Cas9-mediated target gene insertion could be an innovative approach to recombinant protein production for various purposes.

### 4.3. Genome-Edited Birds as Scientific Models

The modification of the avian genome provided a basis for the identification of specific gene functions and the development of genome-edited birds as scientific models. For example, recombination activating gene 1 (*RAG1*) gene was precisely disrupted by CRISPR/Cas9 to obtain chickens lacking mature B and T cells. These chickens could be used to study various lymphocytes in the absence of B and T cells and to study a wide range of diseases, such as cancer and viral infection [192]. In particular, chickens can spontaneously develop ovarian cancer [193]. Thus, *RAG1*-deficient chickens could be an effective model for studying ovarian cancer. In addition, the *GFP* gene was precisely inserted into chicken *DAZL*, a germ cell-specific marker in chicken, to trace germ cells from E2.5 to 1-week post-hatching. Using this model, sex-specific developmental stages and trajectories of chicken germ cells were identified and evolutionary conserved or species-specific genes involved in germ cell development were analyzed [194]. Furthermore, the PR domain zinc finger protein 14 (*PRDM14*) gene, a critical factor for PGC development in mice, was disrupted in PGCs by inserting *eGFP* gene via the CRISPR/Cas9 system to evaluate the important roles of PRDM14 in early chicken development [195]. Similarly, double sex and mab-3-related transcription factor 1 (*DMRT1*) was precisely deleted in chicken, revealing that this gene is one of the most important factors for testis development, while other factors, including sex hormones and *DMRT1* gene networks, are key factors for sex determination [196,197].

The zebra finch is a promising bird for neurobiological studies. The Forkhead box protein 2 (*FOXP2*) mutations in humans lead to developmental verbal dyspraxia (DVD) and lentivirus mediated *FOXP2* gene knockdown in zebra finch results in abnormal speech production [198,199]. Transgenic zebra finches carrying the GFP gene under the control of the human ubiquitin-C promoter were generated and GFP-expressing cells located in the forebrain could be traced and analyzed [38,200]. Gonadal PGCs of zebra finch are heterogenous, and the signaling pathways contributing to their development differ from those of chickens [201,202]. In addition, a retrovirus-mediated immortalized zebra finch fibroblast cell line was established and the *Sox9* gene was knocked out using CRISPR/Cas9 [203]. Collectively, the generation of transgenic zebra finch models would be facilitated by PGC studies, and the utilization of immortalized cell line; such models are expected to contribute substantially to our understanding the mechanism underlying vocal learning in the near future.

## 5. Conclusions

**Figure 2:**
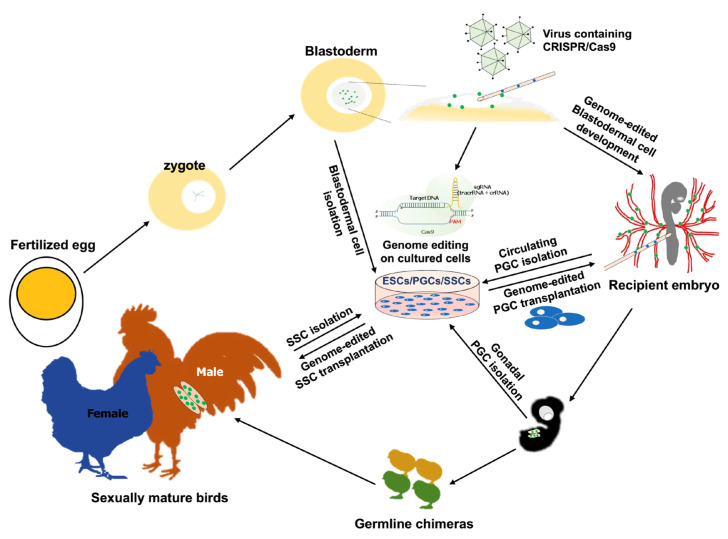
Schematic representation of various strategies for genome-edited bird production. Avian germplasm is found in the zygotic stage as RNA granules and proteins, and a number of germ cells are found in the blastoderm at EGK stage X. In this stage, genome-editing tools, such as CRISPR/Cas9, can be inserted into the subgerminal cavity of the blastoderm with a virus system. The genomes of a small number of germ cells at this stage can be modified, and germline chimera can be formed. Blastoderm cells can be cultured in vitro, and chimera can be established by injecting cultured embryonic stem cells (ESCs) into the subgerminal cavity of recipients, similar to the virus injection system. Primordial germ cells (PGCs) can be isolated from blood vessels of HH stage 13–16 embryos and embryonic gonads of HH stage 28 embryos. The isolated PGCs can be propagated in vitro, and the genome of cultured PGC could be edited using established tools and subsequently transplanted into recipient blood vessels, ultimately forming a germline chimera. From the testes of sexually mature birds, spermatogonial stem cells (SSCs) can be isolated, edited, and implanted into recipient testes to form a germline chimera. The genome-edited germ cells formed from the germline chimera result in a genome-edited bird. The germline cells are shown in green, and cultured/genome-edited cells are shown in blue.

In avian species, gene transfer to early embryos is a potential strategy for genome editing (Figure 2). To date, PGCs are the only avian germline-competent cells that can be cultured in vitro over long time periods and are a reliable means of genome modification. Using viral systems and transplantation methods for other types of cells (e.g., ESCs, SSCs, and iPSCs), the stability and efficiency of germline genome editing are major limitations. For genome editing in a wider variety of bird species, more in-depth studies of these systems are needed, with a focus on efficiency and sustainability. Recently developed genome-editing systems, such as TALEN and CRISPR/Cas, have made it possible to produce genome-edited avian models for various purposes [101,142,150,151]. In particular, the development of avian genetic resources for the control of a wide range of avian diseases, such as avian influenza, should be a focus of future research. The application of genome-editing techniques combined with germline-competent cell-based strategies to a variety of valuable avian species has extensive research applications, and practical applications in the poultry industry. These genome-edited birds will contribute to sustainable agriculture in an eco-friendly manner, providing a basis for reducing the massive culling of virus-infected flocks, improving animal welfare, and increasing production efficiency [204].

## Data Availability

No new data were created or analyzed in this study. Data sharing is not applicable to this article.
